# Limitation of current probe design for oligo-cross-FISH, exemplified by chromosome evolution studies in duckweeds

**DOI:** 10.1007/s00412-020-00749-2

**Published:** 2021-01-14

**Authors:** Phuong T. N. Hoang, Jean-Marie Rouillard, Jiří Macas, Ivona Kubalová, Veit Schubert, Ingo Schubert

**Affiliations:** 1 Leibniz Institute of Plant Genetics and Crop Plant Research (IPK), Gatersleben, 06466 Stadt Seeland, Germany; 2 Present Address: Biology Department, Dalat University, District 8, Dalat City, Lamdong Province Vietnam; 3 Arbor Biosciences, Ann Arbor, MI 48 102 USA; 4 Chemical Engineering Department, University of Michigan, Ann Arbor, MI USA; 5 Biology Centre, Czech Academy of Sciences, Institute of Plant Molecular Biology, CZ 37005 České Budějovice, Czech Republic

**Keywords:** Duckweeds, *Spirodela*, *Landoltia*, *Lemna*, Karyotype evolution, Dual-color oligo-FISH, Microsatellites, Structured illumination microscopy, *Wolffia*, *Wolffiella*

## Abstract

Duckweeds represent a small, free-floating aquatic family (*Lemnaceae*) of the monocot order *Alismatales* with the fastest growth rate among flowering plants. They comprise five genera (*Spirodela, Landoltia, Lemna, Wolffiella,* and *Wolffia*) varying in genome size and chromosome number. *Spirodela polyrhiza* had the first sequenced duckweed genome. Cytogenetic maps are available for both species of the genus *Spirodela* (*S. polyrhiza* and *S. intermedia*). However, elucidation of chromosome homeology and evolutionary chromosome rearrangements by cross-FISH using *Spirodela* BAC probes to species of other duckweed genera has not been successful so far. We investigated the potential of chromosome-specific oligo-FISH probes to address these topics. We designed oligo-FISH probes specific for one *S. intermedia* and one *S. polyrhiza* chromosome (Fig. 1a). Our results show that these oligo-probes cross-hybridize with the homeologous regions of the other congeneric species, but are not suitable to uncover chromosomal homeology across duckweeds genera. This is most likely due to too low sequence similarity between the investigated genera and/or too low probe density on the target genomes. Finally, we suggest genus-specific design of oligo-probes to elucidate chromosome evolution across duckweed genera.

**Supplementary Information:**

The online version contains supplementary material available at 10.1007/s00412-020-00749-2.

## Introduction

Duckweeds comprise together 36 species within five genera: *Spirodela* (2), *Landoltia* (1), *Lemna* (12), *Wolffiella* (10), and *Wolffia* (11). They represent an emerging aquatic crop for feed, food, and biofuel generation, as well as for waste water remediation, due to their fast growth rate, optimal protein profile, and their ability to accumulate minerals and heavy metals (Tippery et al. 2015; Zhao et al. 2015; Appenroth et al. 2017; de Beukelaar et al. 2019; Kaur et al. 2019; Kreider et al. 2019; Yaskolka Meir et al. 2019; Bog et al. 2020; Nahar and Sunny 2020). From the ancient genus *Spirodela* towards the most derived genus *Wolffia*, organismic complexity (many roots *versus* no roots) and size (1.5 cm to less than 1 mm in diameter), genome size (from 160 Mbp to 2.2 Gbp), and chromosome number vary considerably within and between genera (Landolt 1986; Wang et al. 2011; Hoang et al. 2019). Because of these features, duckweeds are an interesting subject for physiological, developmental and evolutionary studies.

The Greater Duckweed, *S. polyrhiza*, was the first duckweed for which a high-quality genome map was generated by integrating results of different approaches such as cytogenomics, optical mapping (BioNano technique), Hi-C conformation study, 454, Illumina, and Oxford Nanopore sequencing platforms (Wang et al. 2014; Cao et al. 2016; Michael et al. 2017; Hoang et al. 2018; Harkess et al. 2020). So far, cytogenetic maps, based on chromosomal localization of ~ 100 anchored BACs, revealed no structural rearrangements between the genomes of seven investigated *S. polyrhiza* accessions of different geographic origin, suggesting a considerable homogeneity between these asexually propagating clones.

*S. polyrhiza* (2*n* = 40) and *S. intermedia* (2*n* = 36), both with a genome size of 160 Mbp/1C, are the only two species of the genus *Spirodela*. Previous cross-FISH with 93 anchored *S. polyrhiza*–specific BAC probes discovered chromosome homeology and several rearrangements between *S. polyrhiza* and *S. intermedia* karyotypes (Hoang and Schubert 2017). These data suggest a considerable infrageneric genome diversity among duckweeds. Cross-FISH with *S. polyrhiza*–specific BAC probes to species of other duckweed genera (*Landoltia punctata, Lemna aequinoctialis, Wolffiella hyalina, Wolffia arrhiza)* yielded only weak and dispersed, but no reliable chromosome-specific signals, even under highly stringent conditions (Hoang 2019).

Cross-FISH with BACs (Lysak et al. 2006; Mandakova and Lysak 2008; Ma et al. 2010) or oligo-probes (Aurich-Costa et al. 2007; Han et al. 2015; Braz et al. 2018; Simonikova et al. 2019; Liu et al. 2020; Xin et al. 2020) were shown to be powerful for comparative chromosome studies, by labeling homeologous chromosomes or chromosome regions of related taxa.

**Fig. 1:**
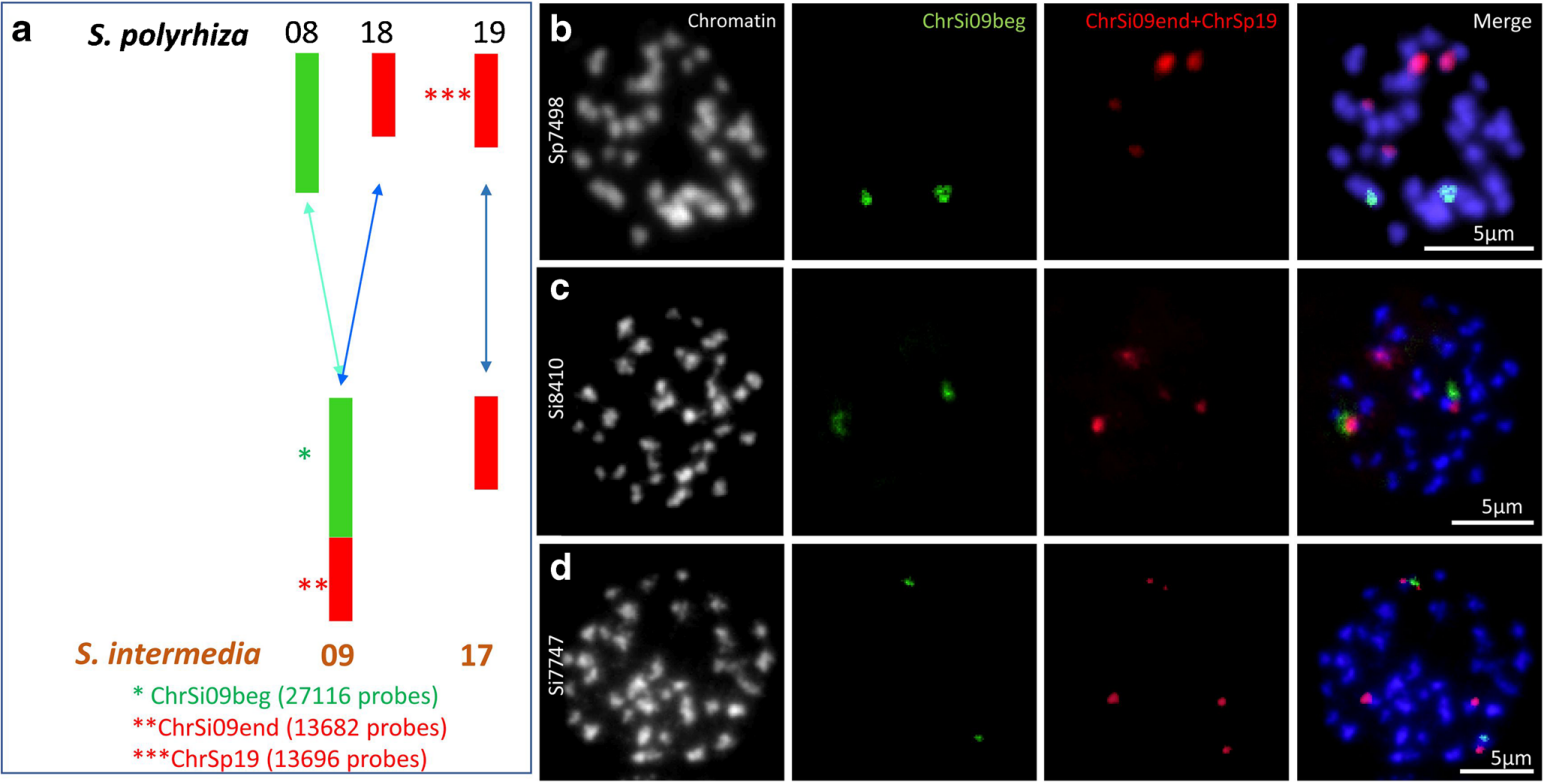
Oligo-FISH confirmed chromosome fusion in *S. intermedia.* **a** Scheme of homeologous chromosomes of *S. polyrhiza* and *S. intermedia*used for oligo-probe design; **b** oligo-probes labeled three different chromosome pairs in *S. polyrhiza*; **c**, **d** the co-localization of green (ChrSi09beg) and red (ChrSi09end) signals which label ChrSp08 and ChrSp18, respectively, confirmed their combination into ChrSi09 of *S. intermedia* (clones 8410 and 7747)

Here, we attempted to apply chromosome-specific oligo-probes for cross-FISH on duckweeds. Oligo-probes were designed for *S. intermedia* chromosome ChrSi09 and *S. polyrhiza* chromosome ChrSp19 (Fig. 1a), based on chromosome-scale sequence assemblies for both species. The former chromosome corresponds to *S. polyrhiza* ChrSp08 and ChrSp18 and the latter one to *S. intermedia* chromosome ChrSi17 (Hoang and Schubert 2017; Hoang et al. 2020). While these oligo-probes hybridized nicely on the original chromosomes and labeled the homeologs of the sister species as well, none or only very weak and dispersed, but no chromosome-specific signals appeared after cross-FISH on mitotic chromosomes of duckweed species of other genera. Blocking of some microsatellite sequences within the oligo-probes, which may cross-hybridize to dispersed repeats of *La. punctata*, did not improve signal specificity. Thus, oligo-FISH across duckweed genera remains a challenge when the density of oligos which find homologous sequences within a distinct region of the target genome falls below a threshold required for a reliable FISH signal and may require a different probe design strategy. Therefore, we suggest to design synteny-based oligo-probes for each genus and to filter out microsatellite-containing oligos for studying chromosome evolution across duckweed genera.

## Material and methods

### Plant material

*S. polyrhiza* (clone 7498), *S. intermedia* (8410 and 7747), *La. punctata* (7260), *Le. aequinoctialis* (clone 2018), *Wa. hyalina* (8640), and *Wo. australiana* (7540) were obtained from Elias Landolt’s collection via BIOLEX (Pittsboro, NC, USA) via K.-J. Appenroth, Friedrich Schiller University, Jena, Germany, and from Rutgers Duckweed Stock Cooperative (New Jersey, USA). Liquid nutrient medium (Appenroth et al. 1996) was used to grow fronds under 16 h white light of 100 μmol m^−^^2^ s^−1^ at 24 °C.

### Probe design

Oligonucleotide probes were designed using Abor Biosciences’ proprietary software. Briefly, target sequences are cut into 43–47 nucleotides-long overlapping probe candidate sequences that are compared to the rest of the genome sequence to check for potential cross-hybridization based on a predicted *Tm* of hybridization. Non-overlapping candidates with no cross-hybridization were blasted against the homeologous *Spirodela* chromosome. Candidates with a single hit on the homeologous chromosome with an *E*-value less than 1E-05 were selected for the final set. This *E*-value cutoff return probes with at least 75% sequence similarity over the entire probe length or a higher similarity over a shorter section of the probe sequence. Probe hybridization to other genera genomes were predicted using the same blast *E*-value cutoff. Probe/target melting temperatures were predicted using the nearest-neighbor model with a 330 mM sodium concentration (2× SSC used in post-hybridization washes).

### Genome sequencing and repetitive DNA analysis

Whole genome shotgun sequencing of *La. punctata* clone 7260 was performed by Admera Health, LLC (South Plainfield, NJ, USA) using a KAPA DNA Library kit (Roche) and Illumina platform generating 2 × 151 nt paired-end reads. The reads were deposited to the European Nucleotide Archive (https://www.ebi.ac.uk/ena) under accession number ERR4463159.

For repeat analysis, the reads were trimmed to 142 nt and quality-filtered. A total of 1.8 million randomly sampled paired reads was then used for repeat identification by similarity-based clustering implemented in the RepeatExplorer pipeline (Novák et al. 2013). The pipeline was run with default parameters except for the similarity search options where masking of low complexity regions was disabled. The resulting clusters containing at least 0.005% of the input reads and thus representing highly or moderately repeated elements were annotated and quantified. Additionally, the TAREAN pipeline (Novák et al. 2017) was employed to specifically search for tandem repeats, using 1.4 million input reads. FISH probes for the satellite LDP_SAT were designed based on the satellite consensus monomer sequence reported by TAREAN. The (partially overlapping) probe sequences were 5’-GCG AAA CTT GCC CGA AAT AGC AAA ATC GCC GTT TCT GGC CTA T-3’ (LDP_SAT-H1) and 5’-CGA AAT AGC AAA ATC GCC GTT TCT GGC CTA TCC GGG GGC CTT TTC GG-3’ (LDP_SAT-H2).

### Mitotic chromosome preparation

Spreading of mitotic chromosomes was carried out according to Hoang and Schubert (2017). In brief, healthy fronds were fixed in fresh 3:1 absolute ethanol:acetic acid for at least 24 h after treatment with 2 mM 8-hydroxyquinoline at 37 °C for 3.5 h. Before and after softening in 2 mL PC enzyme mixture [1% pectinase and 1% cellulase in Na-citrate buffer, pH 4.6] for 90 min at 37 °C, samples were washed twice in 10 mM Na-citrate buffer, pH 4.6, for 10 min each. After softening, samples were transferred on slides and all tissue except the meristem region was removed. Meristems were macerated and squashed in 45% acetic acid. Slides were frozen in liquid nitrogen for 5 min. After carefully removing of coverslips with a razor blade, slides were treated with pepsin [50 μg/mL in 0.01 N HCl] for 5 min at 37 °C, post-fixed in 4% formaldehyde in 2× SSC [300 mM Na-citrate, 30 mM NaCl, pH 7.0] for 10 min, rinsed twice in 2× SSC, 5 min each, dehydrated in an ethanol series (70, 90, and 96%, 2 min each), and air-dried.

### Probe preparation

The *Arabidopsis thaliana* type telomere probe was generated by PCR using TTTAGGG-tetramers without template DNA according to (Ijdo et al. 1991). The product was labeled with Alexa Fluor 488-5-dUTP by PCR (100 ng telomere PCR product in 25 μL reaction mixture), ethanol precipitated (Mandakova and Lysak 2008), dissolved in 250 μL DS20 buffer [50% (v/v) formamide, 20% (w/v) dextran sulfate in 2× SSC, pH 7] at 37 °C for at least 1 h, and stored at – 20 °C (Hoang and Schubert 2017).

MyTags® ChrSi09beg green-labeled probes (ATTO-488) and ChrSi09end/ChrSp19 red-labeled probes (ATTO-594) were obtained from Arbor Biosciences (Ann Arbor, USA). Microsatellite probes were obtained from Eurofins. Lyophilized probes were dissolved in TE buffer [10 mM Tris-HCl, 1 mM Na_2_-EDTA] to a final concentration of 10 pmol/μL, aliquoted, and frozen.

### Oligo-FISH

After adding 100 μL of 70% formamide in 2× SSC on chromosome spreads, they were covered with parafilm, and denatured on a heating plate for 2.5 min at 70 °C. After removing the parafilm, slides were dipped in pre-cooled ethanol series (70, 90, and 96%) for 5 min each on a shaker and air-dried.

Twenty microliters of hybridization mixture (50% formamide, 2× SSC, 20% dextran sulfate with 4 μL of freshly prepared ATTO 488 and/or 2 μL of ATTO 594-labeled probes in DS20 buffer) was used for each slide. The stringency for hybridization was 84.6 and for post-hybridization washing 89.6. In the case of the blocking experiment, all probes (ChrSi09beg and ChrSi09end, ChrSp19 with (GA)_15_ and (GAA)_10_) were pooled together, evaporated under vacuum, and dissolved in 1 μL of ddH_2_O and 15 μL of DS20 buffer. The entire volume was applied onto the slide. Slides were carefully covered by a coverslip to prevent air bubbles inside, and sealed with a line of rubber cement. Chromosome preparations were denatured together with the probes on a heating plate at 70 °C for 3 min and then incubated in a moist chamber at 37 °C for at least 36 h. Post-hybridization washing was carried out as follows: slides were briefly washed in 2× SSC at room temperature to remove the coverslip, then washed under shaking condition at 42 °C for 20 min for 5 min in 2× SSC at room temperature, dehydrated in an ethanol series (70, 90, and 96%, 2 min each), air-dried in the dark, and counterstained with 10 μL DAPI (2 μg/mL in Vectashield).

### Microscopy and image processing

Widefield fluorescence microscopy for signal detection followed Cao et al. (2016). The images were processed (brightness and contrast adjustment only) and merged using Adobe Photoshop software ver.12 × 32 (Adobe Systems).

To analyze the ultrastructure and spatial arrangement of signals and chromatin at a lateral resolution of ~ 120 nm (super-resolution, achieved with a 488 nm laser), 3D-structured illumination microscopy (3D-SIM) was applied using a Plan-Apochromat 63×/1.4 oil objective of an Elyra PS.1 microscope system and the software ZENblack (Carl Zeiss GmbH). Image stacks were captured separately for each fluorochrome using the 405, 488, and 561 nm laser lines for excitation and appropriate emission filters (Weisshart et al. 2016). Maximum intensity projections of whole cells were calculated via the ZEN software. Zoom-in sections were presented as single slices to indicate the subnuclear chromatin structures at the super-resolution level.

## Results

### Probe design for *S. intermedia* and *S. polyrhiza*

A set of 27,116 probes was designed to cover the first 7.79 Mb of *S. intermedia* ChrSi09 (Si09:1-7790000, referred to as ChrSi09beg) and to maintain cross-hybridization capabilities with *S. polyrhiza* ChrSp08. Another set of 13,682 probes was designed against the rest of ChrSi09 (Si09:7790000-12648911, referred to as ChrSi09end), maintaining cross-hybridization capabilities with *S. polyrhiza* ChrSp18. Finally, a set of 13,696 probes was designed to cover the entire *S. polyrhiza* ChrSp19 (ChrSp19:1-3959484) with the ability to hybridize to *S. intermedia* ChrSi17.

As a way to compare probe density along chromosomes and account for potential regions within which probes cannot hybridize to otherwise homeologous chromosomes in species from other genera (for instance due to larger insertions), we defined the Density 100 index (D100) as the probe density of a moving window of a contiguous set of 100 probes expressed in probes/kb of DNA covered by these 100 probes. Each probe set can be described as a collection of D100. Median D100 is used to compare probe set’s potential hybridization to corresponding target chromosomes (Table 1). The three probe sets have D100 medians of 4.53, 3.69, and 5.01 probes per kb on their chromosome of origin, ChrSi09beg, ChrSi09end, and ChrSp19, respectively. The D100 medians for hybridization to reciprocal homeologous *Spirodela* chromosomes are very similar (4.34, 3.47, and 5.24 for ChrSp08, ChrSp18, and ChrSi17, respectively).

**Table 1:** Origin, number, and density of oligo-FISH probes expected to hybridize with homologous/homeologous regions of different duckweed genomes^a^

Origin of probe set	Target species	Actual target region	Number of probes	median D100 (probes/Kb)
ChrSi09beg (green)	*S. polyrhiza*^*b*^ *(2n = 40, 160 Mbp/1C)*	chrSp08:25541-8456612	27116	4.34
	*S. intermedia*^*c*^ *(2n = 36, 160 Mbp/1C)*	chrSi09:109671-77381134	27116	4.53
	*La. punctata*^*d,e*^ *(2n = 46, 424 Mbp/1C)*	Whole genome	2101	
	*Le. minor*^*f*^ *(2n = 42, 836 Mbp/1C)*	chrLem4B:126930-24098945	2052	0.10
		chrLem4A:270877-17346499	1290	0.08
	*Wo. australiana*^*g*^ *(2n = 40, 432 Mbp/1C)*	chrWoa5:88567-22842065	1182	0.06
ChrSi 09end (red)	*S. polyrhiza*	chrSp18:159-5284411	13682	3.47
	*S. intermedia*	chrSi09:7793003-12619724	13682	3.69
	*La. punctata*	Whole genome	Ʃred: 1987	
	*Le. minor*	chrLem16A:67881-15943339	614	0.05
		chrLem16B:62099-18968151	914	0.05
	*Wo. australiana*	chrWoa16:24408-13727754	543	0.05
ChrSp19 (red)	*S. polyrhiza*	chrSp19:30246-3907208	13696	5.01
	*S. intermedia*	chrSi17:43408-3929553	13696	5.24
	*La. punctata*	Whole genome	Ʃred: 1987	
	*Le. minor*	chrLem17B:6671746-15808965	282	0.08
		chrLem20A:2922-9150510	636	0.07
		chrLem20B:112225-7405979	693	0.10
	*Wo. australiana*	chrWoa4:4039-23215234	714	0.08

^a^For inter-genus hybridization > 75% similarity is assumed

^b^Hoang et al. (2018) for genomic data

^c^Hoang et al. (2020) for genomic data of *S. intermedia* clones 7747 and 8410 see European Nucleotide Archive (ENA) under PRJEB35514 and PRJEB35634, respectively. Raw reads can be obtained from EBI ENA accession numbers PRJEB33624 (PacBio, *S. intermedia* 7747), ERR3829756 (Illumina, *S. intermedia* 8410), and ERR3957957-ERR3957958 (Oxford Nanopore, *S. intermedia* 8410).

^d^For genomic data, see European Nucleotide Archive (https://www.ebi.ac.uk/ena) under accession number ERR4463159.

^e^For Landoltia, the probe values are probably overestimated due to “off-target” hits, because no chromosome assemblies are available.

^f^Because no genomic data are available for *Le. aequinoctialis*, the genomic data for *Le. minor* lemna.org were used

^g^Michael et al. (2020) bioRxiv https://doi.orh/10.1101/2020.03.31.018291 for genomic data

We also computed the theoretical *Tm* value of each probe hybridized to its target in several duckweed genomes (Fig. S5). The medians of the *Tm* distributions for hybridization of the three probe sets to their sequences of origin are around 76–77 °C, Fig. S5). These median *Tm* values drop by about 10 °C when computed for the entire probe sets hybridizing to the homeologous *Spirodela* chromosomes due to sequence divergences between the two species. For *Landoltia, Lemna,* and *Wolffia*, the probe number was strongly reduced to those probes that are expected to hybridize stably. Therefore, the *Tm* values drop less than in the intra-genus comparison.

Probes designed from ChrSi09end and ChrSp19 were synthesized as a single set and labeled separately from probe designed from ChrSi09beg, enabling two-color hybridizations.

### Oligo-cross-FISH confirmed “chromosome fusion” in *S. intermedia*

Using 93 BACs anchored in the *S. polyrhiza* genome, and a suitable BAC pooling system, a cytogenetic map for *S. intermedia* clone 8410 has been established (Hoang and Schubert 2017). At first, we designed oligo-probes to confirm the evolutionary “fusion” of ChrSp08 and ChrSp18 into ChrSi09 (or the split of ChrSi09 into ChrSp08 and ChrSp18), as previously found by cross-FISH with six ChrSp08 BACs (013I04, 006P24, 032L08, 034K03, 004E01, and 006L17) and three ChrSp18 BACs (026D06, 037B13, and 029K19) (Hoang and Schubert 2017).

In order to test the specificity of the synthetic oligo-probe sets, they were hybridized to chromosome spreads of *S. polyrhiza* (clone 7498) and *S. intermedia* (clones 8410 and 7747). These probes labeled the corresponding three chromosome pairs of *S. polyrhiza* (Fig. 1b) and their homeologous counterparts of *S. intermedia* (clones 8410 and 7747), (Fig. 1c, d), confirming that ChrSp08 and ChrSp18 together correspond to ChrSi09.

### Oligo-cross-FISH to other duckweed species

**Fig. 2:**
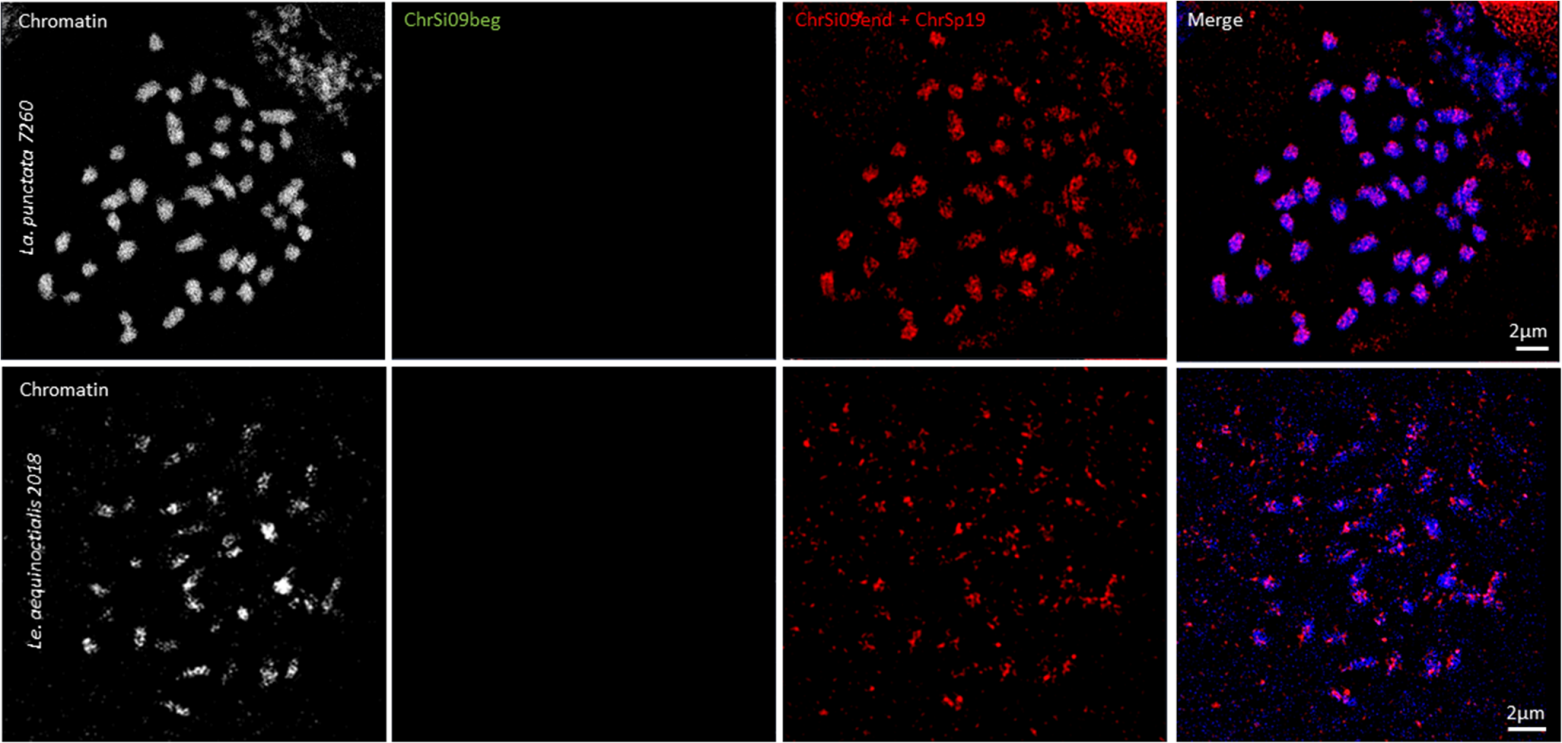
Oligo-FISH with *S. polyrhiza* chromosome–specific probes on *La. punctata* (upper panel) and *Le. aequinoctialis* (lower panel). No green signals, but dispersed red signals were identified by spatial structured illumination microscopy (3D-SIM)

After proving the chromosome specificity of oligo-probes in their species of origin and successful cross-FISH to homeologous *Spirodela* chromosomes*,* the same oligo-probe sets were applied to chromosome spreads of species of the other four duckweed genera. The studied species were (with increasing phylogenetic distance to the genus Spirodela): *La. punctata* clone 7260 (2*n* = 46; 424 Mbp/1C), *Le. aequinoctialis* clone 2018 (2*n* = 42; 452 Mbp/1C)*, Wa. hyalina* clone 8640 (2*n* = 40; 1234 Mbp/1C), and *Wo. australiana* clone 7540 (2*n* = 40; 432 Mbp/1C) (Hoang et al. 2019). No signals were detectable with the oligo-probe set specific for ChrSi09beg on either of the species, even not when structured illumination microscopy (SIM) was applied to achieve super-resolution. The probe specific for ChrSi09end and ChrSp19 generated dispersed signals over nearly the entire chromosome complements of *La. punctata* (Figs. 2 and S1), and *Le. aequinoctialis.* No signals were detectable on chromosomes of *Wa. hyalina* and *Wo. australiana*.

### Dispersed microsatellite sequences in *Landoltia* and *Lemna* target genomes apparently give rise to cross-FISH signals by the *Spirodela*-derived oligo-probes

The dispersed signals of the ChrSi09end/ChrSp19 probe set on *La. punctata* and *Le. aequinoctialis* chromosomes suggest that some oligos are similar to dispersed repetitive sequences within the genomes of the two species. Indeed, some oligo-probe sequences contain for instance (GA)_n_ and (GAA)_n_ microsatellite motifs (Table 2). A randomly sampled fraction of the Illumina reads was used to identify *La. punctata* repetitive elements employing RepeatExplorer and TAREAN pipelines (Novák et al. 2013; Novák et al. 2017). The analysis revealed a relatively small proportion of highly and moderately repeated sequences in *La. punctata* (38% of the genome), with the prevalence of LTR-retrotransposons (20.9%) and tandem repeats (4.7%). The tandem repeats mainly consisted of microsatellite motifs (GA)_n_ and (GAA)_n_.

**Table 2:** Examples of oligo-sequences containing microsatellite **(GA)**_**n**_ and **(GAA)**_**n**_ motifs

ChrSp-specific probe	Oligo-sequences contain microsatellite motifs
ChrSp 08	CCACG**CT**CAAGCAA**GAA**CAA**GA**CCCTTT**GA**G**GAAGAAGAAGAA**AC
	A**GAAGAAGAAGAA**GCAATGGTATCCATG**GAA**T**TTC**T**TTC**ACCTAC
	GGG**GAA**AT**TTC**TGAGGATTT**TTC**TTGCTCTCC**TTCTTCTTCTTC**T
	**GAAGAAGAAGAA**ATAATGGTGCAGCTGG**TTC**GCTG**TTC**ATCTTAG
	**GAAGAAGAAGAA**AGCATTG**GAA**A**GAA**ATCTTAG**GAA**GGTCGATGG
	A**GAGAGAGAGAGAA**GTA**GA**TAGGT**GA**TGGCAA**GA**TGGTCGTTGCG
	T**CTCTCTCTCTCT**AA**GA**TC**CTCT**TTGGTACATG**GAA**GGTACCGTG
	A**GAGAGAGAGAGAA**ATGCTCAA**GA**CACATTT**GA**C**TTC**TGCGTATG
	**GAGAGAGAGAGA**GCATAGGGG**TTC**AATGTCTAGT**GA**CTA**GA**TGCC
	**GAGAGAGAGAGA**TTATCAT**GA**TG**CT**GT**CT**TATGTCAATCAAAGGC
ChrSp 18	A**GAAGAAGAAGAA**GAGGATA**GAA**CCGTTTGACGACCTCTCT**TTC**C
	C**TTCTTCTTCTTC**TGGG**TTC**GATCAGTCTGTGCGT**GAA**GGGGTAC
	AG**GA**G**GA**GCA**GAA**ACCTA**GAAGAAGAAGAA**GCTCACG**TTCTTC**CG
	C**TTCTTCTTCTTC**ATAAGTCTACCGGCCG**GA**TAACCA**GAA**GT**GA**G
	A**GAGAGAGAGAGA**GTTAAATAG**GA**CGTACAA**TTC**CTAC**GAA**CCAA
	**CTCTCTCTCTCT**ACA**TTC**TGGTGCATC**GA**CACA**GAGA**TA**GA**TCCT
	T**CTCTCTCTCT**C**TTC**GTTAA**GAA**AACATCTTGTTG**GA**CTA**GA**CGT
ChrSp 19	AGTATCATCAAGT**GAAGAAGAAGAA**TGCTTGACACAGGCTCG**TTC**
	AA**GAAGAAGAAGAA**GCAATTG**GAA**AAA**GAA**CTCGCGGCTGTCTGA
	AAATCCAGC**GA**TG**GAA**CCATCATG**GA**GGTCC**TTCTTCTTCTTC**TT
	**GAGAGAGAGAGAGA**TGTAAATAAGTCAACTGGT**GA**T**GA**TGCCACT
	**CTCTCTCTCTCT**A**GAA**TACCATGCA**GA**TCAG**GAA**TGTGCAAAACC
	A**GAGAGAGAGAGAA**TTTGTGTGCAGT**GA**CC**GA**GTCCTTACTCTCT
	A**GAGAA**AGTACA**GAGAGAGAGAGA**TT**GA**GGCACCT**GAAGA**CCGGC

**Fig. 3:**
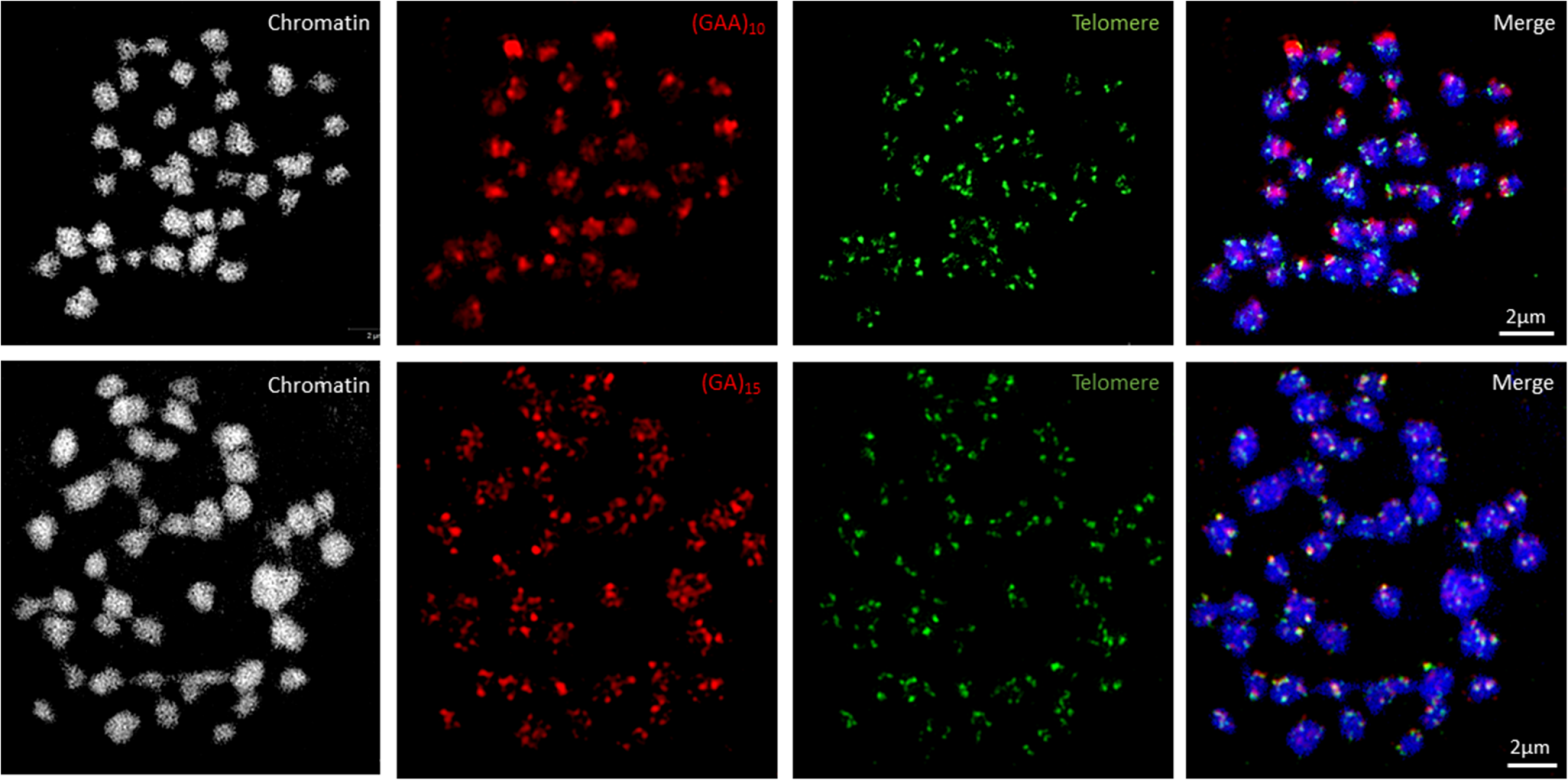
Distribution of GAA and GA microsatellite signals on *La. punctata* chromosomes. GAA (upper panel), GA (lower panel) microsatellite probes, and telomere repeats (TTTAGGG); imaged by 3D-SIM

In order to check the chromosomal distribution of these microsatellite motifs on *La. punctata*, we performed FISH with labeled (GA)_15_ and (GAA)_10_ sequences as probes. Both probes hybridized to all chromosomes (Figs. 3 and S2) mostly in terminal regions as shown by the partial overlap with signals for the *Arabidopsis*-type telomere sequence repeat (TTTAGGG_n_). One *La. punctata* satellite repeat revealed a monomer length of 138 bp and an estimated abundance of 0.21% of the genome (LDP_SAT1). FISH with two partly overlapping oligos of 42 nt (H1) and 47 nt (H2) of this GC-rich satellite sequence yielded similar signals as were obtained with labeled (GA)_15_ and (GAA)_10_ probes (Fig. S3).

To reduce binding of oligos to dispersed repeats of *La. punctata*, unlabeled (GA)_15_ and (GAA)_10_ sequences were added in excess (50 pmol of each microsatellite/20 pmol total labeled oligos of each chromosome/slide) to the probe. In spite of a reduction of dispersed signals, no specific labeling of distinct *La. punctata* chromosomes was recognizable (Fig. S4). Apparently, several further dispersed repeats of the target genomes match with *Spirodela-*derived oligos from ChrSi09end and/or ChrSp19 and yield dispersed signals on the chromosomes of *La. punctata* and *Le. aequinoctialis.*

### Computational probe mapping to other duckweed genera

To predict the ability of the *Spirodela-*derived oligos to hybridize to chromosomes of other duckweed genera, probe sequences were blasted against the *Le. minor* (tetraploid) and *Wo. australiana* genomes. *Le. minor* is used here as a surrogate for *Le. aequinoctialis*, of which the genome is not yet sequenced. Best Blast hits with sequence similarity deemed enough to generate a stable hybridization (> 75% similarity) were sorted by chromosomes (Table S1). Probes designed from ChrSi09beg preferentially match *Le. minor* chromosomes ChrLemA4 and B4 (1290 and 2055 hits, respectively), and *Wo. australiana* chromosome ChrWoa5 (1188 hits). Similarly, probes designed from ChrSi09end match *Le. minor* chromosomes ChrLemA16 and B16 (614 and 915 hits, respectively) and *Wo. australiana* chromosome ChrWoa16 (543 hits). Finally, probes designed from ChrSp19 match *Le. minor* chromosomes ChrLemB17, A20, and B20 (282, 636, and 693 hits, respectively) and *Wo. australiana* chromosome ChrWoa04 (715 hits). To acquire sequence information for evaluating similarities of *Spirodela*-based oligo-probes to their targets in the *La. punctata* genome, shotgun Illumina sequencing of the *La. punctata* genome (clone 7260) was performed and yielded 301.7 million pairs of raw Illumina reads (2 × 151 nt, ENA accession number ERR4463159), corresponding to a ~ 215-fold genome coverage. The oligo-probe sequences were blasted against unassembled *La. punctuata* genome reads, generating 2010 and 1987 hits for ChrSi09beg and ChrSi09end/ChrSp19 probe sets, respectively. Due to the absence of an assembled genome for *La. punctuata*, likely the number of probes hybridizing to homeologous chromosomes is overestimated since 5 to 15% of all hits on *Le. minor* and *Wo. australiana* genomes do not land on preferential chromosomes (Table S1). The small number of probes able to hybridize to *Le. minor* and *Wo. australiana* genomes and the fact they are spread across multiple chromosomes lead to very low median D100 probe densities (0.05 to 0.1 probes/kb, see Table 1, Fig. S6) compared to hybridizations between the *Spirodela* species. Most likely the same is true for *La. punctuata*.

## Discussion

Our oligo-cross-FISH experiments yielded strong and specific signals only within the genus *Spirodela*. The absence of chromosome-specific signals after cross-FISH across the genus border is likely due to the low number of probes with enough sequence similarity to achieve a stable hybridization between the probe and the chromosomes of the tested species. Similarly, while probes of > 90% similarity equally well labeled homeologous chromosomes of the allotetraploid switchgrass *Panicum virgatum*, probes of < 75% similarity yielded virtually no signals on homeologs (Jiang 2019).

An alternative or additional explanation might be a too large distance between probes hybridizing to the target chromosome to generate a detectable signal. We computationally demonstrated that the number of probes able to stably hybridize to chromosomes across the genus border is strongly reduced compared to intra-genus hybridization. This is leading to a 40- to 80-fold reduction in probe density along the target sequence. It is likely that the reduced number of probes able to hybridize to homeologous chromosome regions of species across the genus border is too distantly located along the target chromosomes to generate detectable chromosome-specific signals. Although successful oligo-FISH with various densities (0.1–0.5 oligos/kb; Jiang 2019) has been reported for different plant species, and Song et al. (2020) found even 0.052 oligos/1 kb of the target chromosome 4D of wheat sufficient for reliable chromosome-specific labeling, such low density of oligo-sequences did not generate reliable FISH signals in cross-hybridization between duckweed genera which apparently have a less dense chromosome structure than wheat. Similarly, Simonikova et al. (2019) found in banana chromosome complements that a density of 0.8 oligos/kb did no longer label target chromosome regions contiguously. Albeit also technical details of oligo-FISH approaches could influence the results, sequence similarity and probe density have to be optimally adjusted for each case of oligo-cross painting.

Because a bioinformatic comparison of the different probe sets did not reveal any differences that could explain why only the ChrSi09end/ChrSp19 probe set leads to such dispersed signal and not the ChrSi09beg probe set, possibly, the ATTO-488-labeled probe set signal was weaker than the one from the ATTO-594-labeled set and therefore yielded no detectable signals across the genus border.

Probes used in the present study were designed using only *Spirodela* species genomes to predict and exclude sequences capable of forming non-specific hybridizations. Our observation of dispersed signals in *Landoltia* and *Lemna* and lack of specific chromosome labeling across the genus border support the need to also include genomic sequences from species of other genera and chromosome synteny data during the probe selection process. Unassembled reads from a relatively inexpensive shallow-depth shotgun sequencing should provide enough information to select probes that can produce strong and specific signals in multiple genera, granted that at least one species included in the study has been sequenced and fully assembled.

**Fig. 4:**
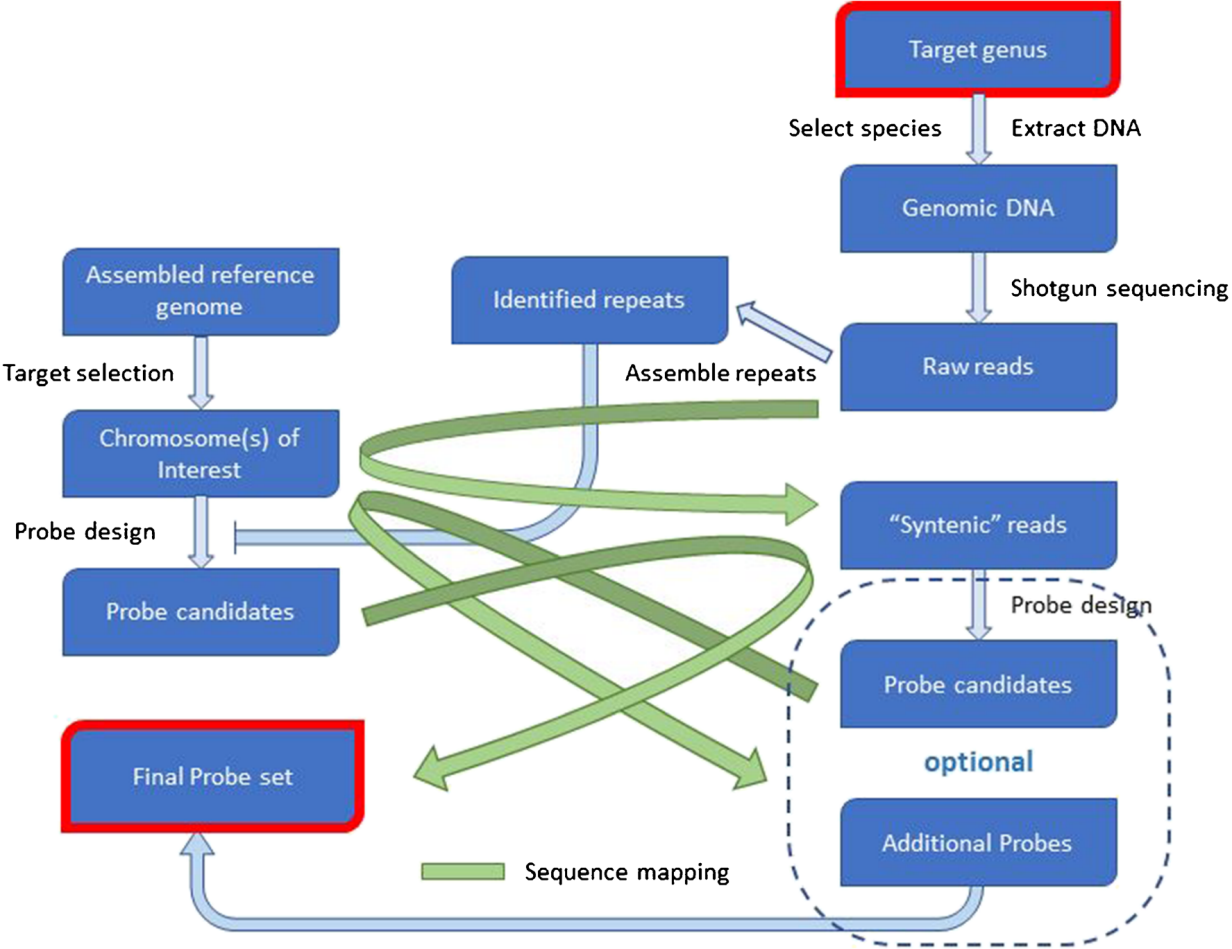
Proposed workflow for designing probes for oligo-FISH across genera

We propose the following workflow to design FISH probes for studies across genera (Fig. 4). The genomic DNA from a representative species for each target genus should be shotgun-sequenced. A shallow 5–10 × coverage should provide enough data to identify the most common repeats present in that genome. The reads should also be mapped to the reference species genome. Reads mapping preferentially to the chromosome(s) of interest should be selected as reads from syntenic regions. Probes should be designed against the chromosomes of interest from the reference species. Candidate probes should be checked for lack of cross-hybridization against the repeat sequences obtained from the newly sequenced species. Candidate probes should be also mapped to the syntenic reads to select probes with greater than 85% homology with the other genus sequence. If more probes are needed, an optional probe design could be done using the syntenic reads as input. These additional candidate probes should be compared to the reference genome to ensure they are specific to the intended reference target chromosome and can hybridize to species from both genera. More elaborated design workflows could involve assembling overlapping reads into larger contigs to expand the probe design space in the newly assembled genome. This may be helpful in designing probes for phylogenetically distant genera.

## Conclusions

Oligo-probes (as well as BACs) yielded chromosome-specific FISH signals within duckweed species of the same genus, but not across genus borders, apparently because of too low density of oligos sufficiently similar to the target chromosome sequences. Minisatellite motifs within the probes may yield dispersed FISH signals, when abundant in the target genome. Oligos containing such motifs should be filtered out. If no assembled genomes are available for the genus of the target species and oligo-FISH across the genus border does not give chromosome-specific results, oligo-probes should be designed from shotgun sequences based on synteny with a related genus. Suitability of probes should be validated by FISH on homologous chromosomes before applied for congeneric karyotyping and identification of homeologous/rearranged chromosomes of congeneric species.

## Supplementary information

ESM 1 (PDF 880 kb)
