# Supplementary material for: Limitation of current probe design for oligo-cross-FISH, exemplified by chromosome evolution studies in duckweeds
Source: Chromosoma. 2021 Jan 14;130(1):15–25. doi: 10.1007/s00412-020-00749-2 (PMC7889562; doi:10.1007/s00412-020-00749-2)
Supplement: Supplementary file 1 — (PDF 880 kb) [file 412_2020_749_MOESM1_ESM.pdf]

Limitation of current probe design for oligo-cross FISH, exemplified by chromosome evolution studies in duckweeds

Phuong TN Hoang[1,2], Jean-Marie Rouillard[3,4], Jiří Macas[5], Ivona Kubalová[1], Veit Schubert[1], Ingo Schubert[1*]

[1]Leibniz Institute of Plant Genetics and Crop Plant Research (IPK), Gatersleben, D-06466 Stadt Seeland, Germany

[2]Permanent address: Biology Department, Dalat University, District 8, Dalat City Lamdong Province, Vietnam

[3]Arbor Biosciences, Ann Arbor, MI 48 102, USA

[4]Chemical Engineering Department, University of Michigan, Ann Arbor, USA

[5]Biology Centre, Czech Academy of Sciences, Institute of Plant Molecular Biology, České Budějovice, CZ 37005, Czech Republic

* Corresponding author: schubert@ipk-gatersleben.de, Tel: +49 394825239

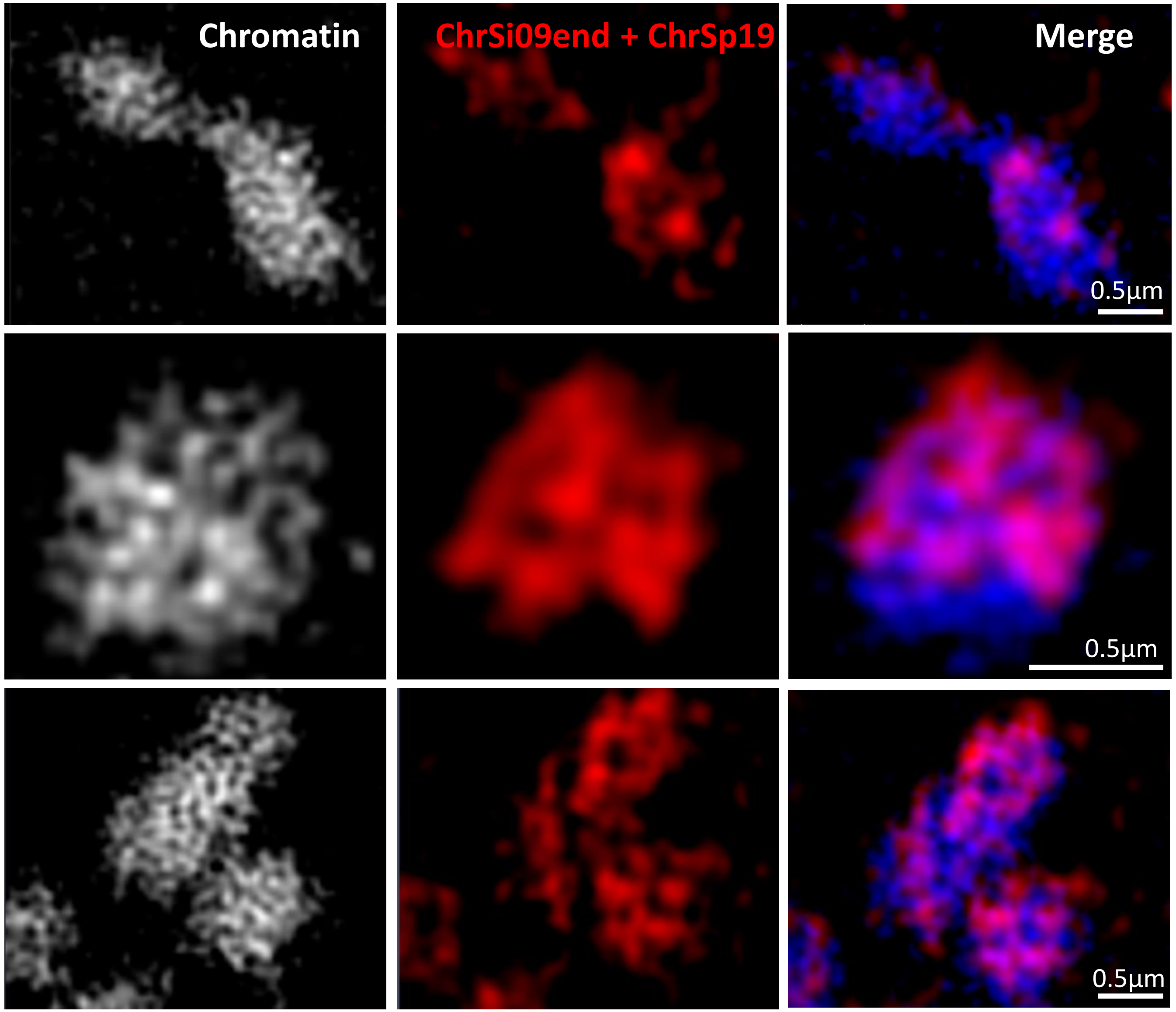

Figure S1: Oligo-probe ChrSi09end + ChrSp19 labels *La. punctata* chromosomes entirely. Imaged by 3D-SIM.

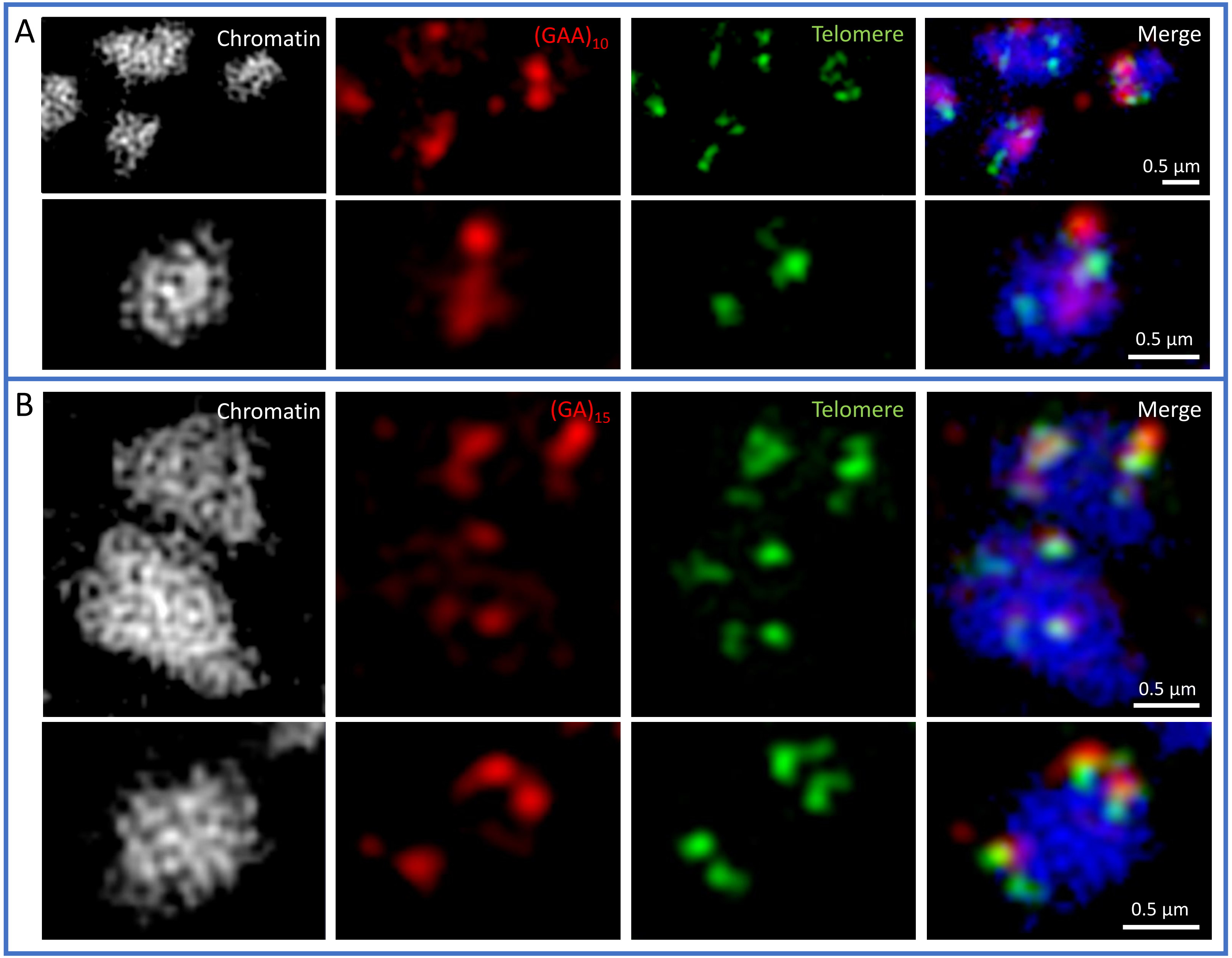

Figure S2: GAA and GA microsatellite signals on *La. punctata* chromosomes are close to the chromosome ends. Microsatellite motif GAA (A) or GA (B) and telomere repeats(green), imaged by 3D-SIM.

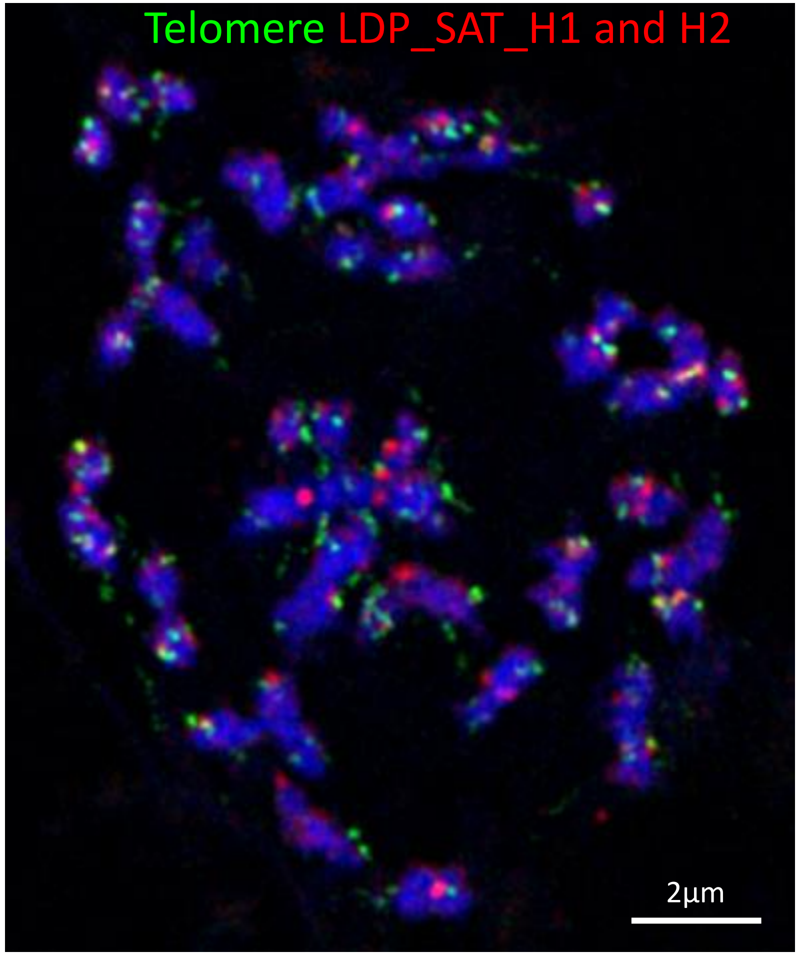

Figure S3: FISH signals of LDP_SAT1 sequences H1 and H2 on *La. punctata* chromosomes.

ATGCGAAACTTGCCCGAAATAGCAAAATCGCCGTTTCTGGCCTATCCGGGGGCCTTTTCGGGCGCCGTCGACCTCTCGGGGAGCTTTGGCGCCGGGGCGGAGCGGCTGGAGGCCGCGGACCCGGATTCCGGGCCAGAA

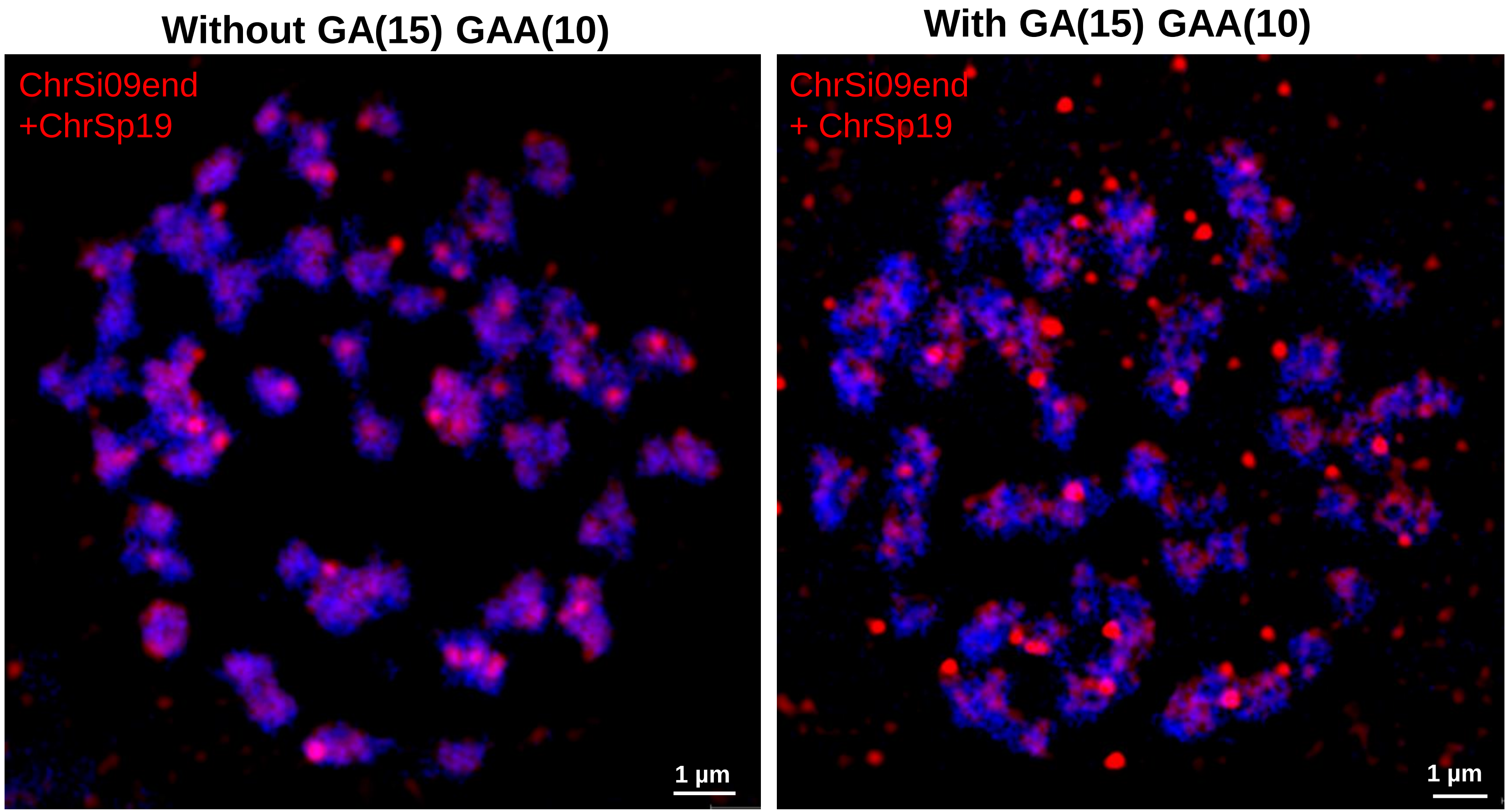

Figure S4: Oligo-FISH with ChrSi09end and ChrSp19 probes and excess of unlabeled microsatellites GA and GAA on *La. punctata* chromosomes. Blocking of oligos that match with GA and GAA microsatellites did not lead to chromosome-specific FISH signals (right panel); imaged by 3D-SIM.

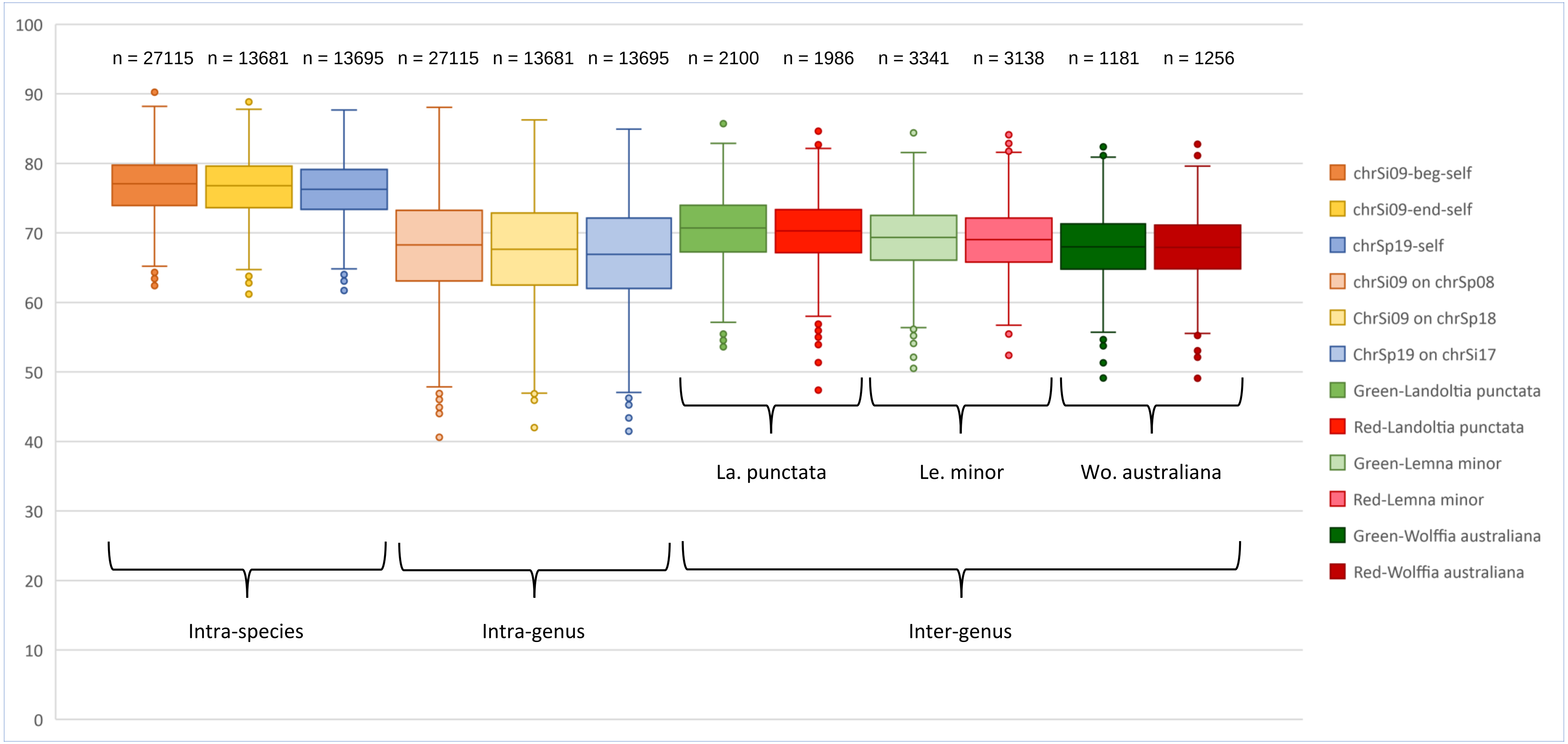

Figure. S5 Probe *Tm* distributions for intra-species, intra- and inter-genus hybridizations. Probe *Tm* values (°C) are calculated for each probe hybridizing to its target in the denoted genome. The number of probes included in each distribution is noted above its respective box plot. ChrSi09end and ChrSp19 probes were combined as the "red" probe set and probes from ChrSi09beg form the "green" probe set. 60 million unassembled reads were used to generate *La. punctuata* data while assembled genomes were used for *Le. minor* and *Wo. australiana*. Since probes included in the distributions for Landoltia, Lemna, and Wolffia (but not those for intra-genus comparisons) were reduced based on their ability to hybridize stably, the probe numbers (Table 1, Fig. S6) reflect the phylogenetic distances more strongly than the *Tm* value distributions. Nevertheless, with equal filtration threshold, the median *Tm* values for the considered probes reflect the phylogenetic distances between *Spirodela*, *Landoltia*, *Lemna*, and *Wolffia* genera.

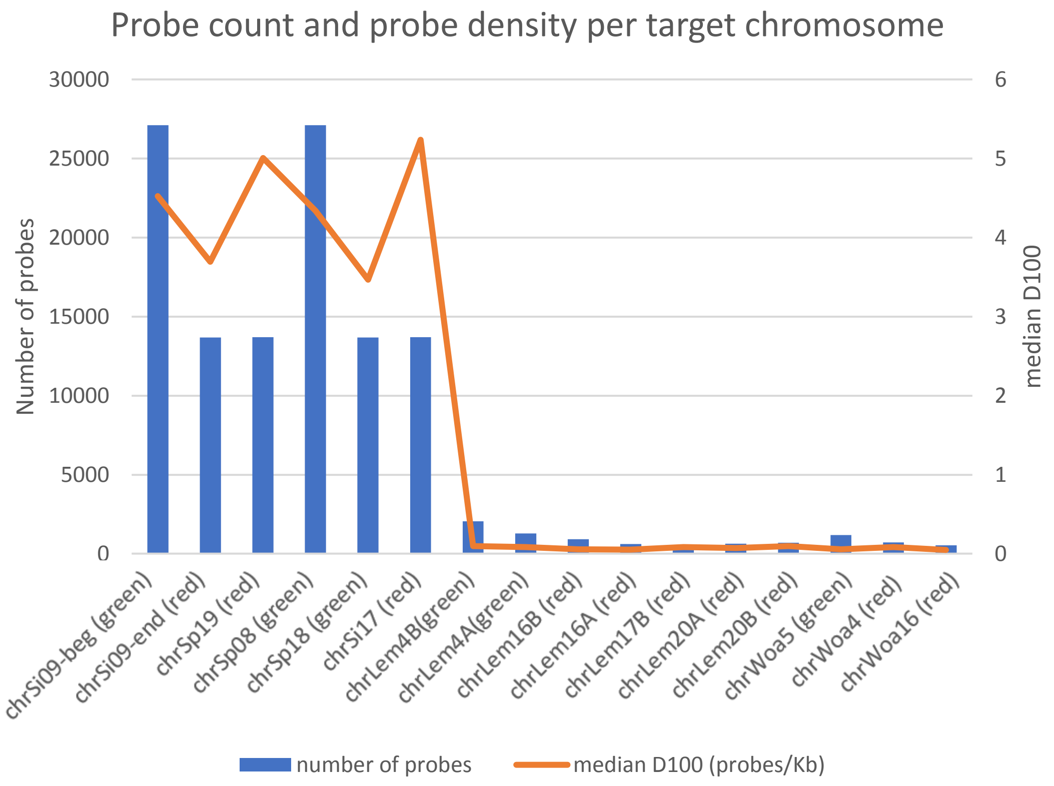

Figure S6: Probe count and probe density per target chromosome according to table 1.
(green) = probes from ChrSi09beg; (red) = probes from ChrSi09end + ChrSp19.

Table S1. Chromosomal distribution of oligo probes derived from *S. intermedia* (ChrSi09beg and ChrSi09end) and *S. polyrhiza* (ChrSp19) chromosomes with >75% similarity to sequences within the target genomes

**ChrSi09beg on *Le. minor***		**ChrSi09end on *Le. minor***		**ChrSp19 on *Le. minor***		**ChrSi09beg on *Wo. australiana***		**ChrSi09end on *Wo. australiana***		**ChrSp19 on *Wo. australiana***	
1B	12	1B	9	**7A**	**282**	chr1	11	chr1	12	**chr4**	**715**
8B	6	8B	6	17B	2	chr10	15	chr10	14	chr5	18
10B	4	10B	2	14B	2	chr11	7	chr11	8	chr6	12
7B	21	7B	4	13A	3	chr12	8	chr12	11	chr7	3
13B	2	13B	5	3B	6	chr13	10	chr13	11	chr8	2
9B	4	9B	5	18B	1	chr14	16	chr14	3	chr9	13
3A	2	3A	3	15A	3	chr15	17	chr15	4	***Total***	***763***
6A	1	6A	1	14A	2	chr16	10	**chr16**	**543**	***on target***	***90%***
15B	5	**16B**	**915**	12A	1	chr17	5	chr17	5	***off target***	***10%***
8A	8	15B	2	19B	5	chr18	8	chr18	4		
2B	8	8A	2	**20A**	**636**	chr19	4	chr19	5		
4A	4	2B	5	21B	12	chr2	20	chr2	4		
**11B**	**1290**	4A	1	17A	4	chr20	2	chr20	4		
9A	5	11B	9	1A	3	chr3	14	chr3	6		
10A	3	9A	1	**20B**	**693**	chr4	18	***Total***	***634***		
16A	1	10A	1	19A	1	**chr5**	**1188**	***on target***	***90%***		
12B	6	**16A**	**614**	21A	16	chr6	7	***off target***	***10%***		
7A	3	12B	4	5B	3	chr7	19				
14B	7	***Total***	***1589***	2A	10	chr8	9				
3B	8	***on target***	***95%***	4B	10	chr9	5				
18B	1	***off target***	***5%***	5A	5	***Total***	***1393***				
15A	4			6B	1	***on target***	***85%***				
11A	2			***Total***	***3290***	***off target***	***15%***				
14A	3			***on target***	***95%***						
12A	4			***off target***	***5%***						
19B	2										
20A	7										
17A	1										
1A	10										
20B	5										
5B	2										
2A	19										
**4B**	**2055**										
5A	5										
6B	5										
Total	***3525***										
on target	***95%***										
off target	***5%***										

On target: extended syntenic regions
